# Association of Body Water Balance, Nutritional Risk, and Sarcopenia with Outcome in Patients with Acute Ischemic Stroke: A Single-Center Prospective Study

**DOI:** 10.3390/nu16132165

**Published:** 2024-07-08

**Authors:** Takayoshi Akimoto, Kenta Tasaki, Masaki Ishihara, Makoto Hara, Hideto Nakajima

**Affiliations:** Division of Neurology, Department of Medicine, Nihon University School of Medicine, 30-1 Oyaguchi-Kamicho, Itabashi-ku, Tokyo 173-8610, Japannakajima.hideto@nihon-u.ac.jp (H.N.)

**Keywords:** bioelectrical impedance analysis, acute ischemic stroke, outcome, overhydration, sarcopenia, nutritional risk

## Abstract

In the present study, we examined the inter-relationships between body water balance, nutritional risk, sarcopenia, and outcome after acute ischemic stroke (AIS) in patients who were living independently. We defined abnormal body water balance as overhydration, with an extracellular fluid/total body water ratio > 0.390. A geriatric nutritional risk index (GNRI) < 98 was considered low GNRI. Sarcopenia was defined according to the 2019 Asian Working Group for sarcopenia criteria. Poor outcome was defined as a modified Rankin scale (mRS) score ≥ 3 at discharge. Among 111 eligible patients (40 females, median age: 77 years), 43 had a poor prognosis, 31 exhibited overhydration, 25 had low GNRI, and 44 experienced sarcopenia. Patients with poor outcomes had significantly higher National Institutes of Health Stroke Scale (NIHSS) scores, which were significantly more common with overhydration, low GNRI, and sarcopenia (*p* < 0.001 for all). Concomitant overhydration, low GNRI, and sarcopenia were associated with poorer outcomes. In multivariate analysis, overhydration [odds ratio (OR) 5.504, 95% confidence interval (CI) 1.717–17.648; *p* = 0.004], age (OR 1.062, 95%CI 1.010–1.117; *p* = 0.020), and NIHSS score (OR 1.790, 95%CI 1.307–2.451; *p* < 0.001) were independent prognostic factors for poor outcome. The results indicated that the combination of overhydration, low GNRI, and sarcopenia predict poor outcomes following AIS. Overhydration was particularly associated with poor outcomes.

## 1. Introduction

Acute ischemic stroke (AIS) is a leading cause of death and disability worldwide [1,2]. Because 35% of all patients with post-stroke disability are institutionalized, improving motor function and outcomes after stroke is important [1]. Sarcopenia is a muscle disease that adversely impacts independence and daily living [3,4]. It results in poor functional prognosis in patients with AIS and sarcopenia [5,6,7]. According to the consensus guidelines of the European Working Group on Sarcopenia in Older People 2 [4] and the 2019 Asian Working Group for Sarcopenia [3], sarcopenia is defined as a reduction in muscle strength or physical performance as well as low muscle mass or quality. Muscle strength and physical performance may be evaluated using grip strength, chair-stand test, and gait speed [3,4], whereas skeletal muscle mass is evaluated using dual-energy X-ray absorptiometry or bioelectrical impedance analysis (BIA) [3,4]. In addition to skeletal muscle mass, BIA can simultaneously measure body composition and body water balance. In humans, most water is distributed as intracellular water (ICW) and extracellular water (ECW), and total body water (TBW) is the sum of the two. The ECW/TBW ratio indicates body water balance and is controlled within a certain range [8,9,10,11,12,13,14,15]. An increase in ECW/TBW indicates overhydration [8,9,10,14]. Furthermore, the ECW/TBW ratio is positively associated with cellular damage due to sarcopenia [16] and undernutrition [17,18]. In addition, the complications of sarcopenia [5,6,7] and undernutrition [19,20,21,22] are associated with poor stroke prognosis. Studies in acute care hospitals have shown a relationship between the ECW/TBW ratio and stroke during post-stroke dehydration [23], assessment of fluid replacement volume [24], and stroke subtype (ischemic or hemorrhagic) [25]. Although the relationship of the ECW/TBW ratio with patient outcomes has been investigated in sepsis [26], cancer [17], and heart failure [27], to date, no study has evaluated the relationship between the ECW/TBW ratio and stroke outcomes in acute care hospital. This study aimed to determine the usefulness of BIA for patients with AIS in acute care hospitals, the inter-relationship between nutritional risk, sarcopenia, and body fluid balance/overhydration, and their association with outcomes in AIS patients who were living independently before AIS.

## 2. Materials and Methods

### 2.1. Patient Selection and Data Collection

This single-center prospective study included patients with AIS admitted to the Department of Neurology at the Nihon University School of Medicine (Tokyo, Japan) between August 2021 and December 2022. The Department of Neurology accepted patients with AIS who were not indicated for alteplase or endovascular treatment. AIS diagnosis was based on the presence of lesions on diffusion-weighted magnetic resonance imaging obtained within 7 days of the onset of new neurological symptoms. The exclusion criteria included rejection to provide informed consent, inadequate BIA, and a pre-AIS modified Rankin scale (mRS) score ≥ 3. Patients with pacemakers, and those undergoing hemodialysis were excluded due to the potential adverse effects of BIA on pacemakers and the possibility of significant changes in body water balance due to hemodialysis [28]. Additionally, patients who were not able to stay still during BIA, those who were in an isolation ward for long durations due to severe acute respiratory syndrome coronavirus 2 infection, and those who died between the time of study consent and BIA were excluded. All eligible patients or their legal guardians provided written informed consent before enrolment.

Blood sampling data (albumin, creatinine, *C*-reactive protein (CRP), *N*-terminal-pro-brain natriuretic peptide (NT-pro-BNP), and glycated hemoglobin (HbA1c)) were collected at the time of admission. NT-pro-BNP is considered a marker of congestive heart failure [29]. In the present study, an NT-pro-BNP level of 500 pg/mL or higher in patients was considered high [29]. To assess the severity of AIS at admission, the National Institutes of Health Stroke Scale (NIHSS) score was used [30]. It consists of 15 items to evaluate neurological symptoms, each of which is scored from 0 to 2 or 0 to 3. The total score (maximum score of 42) is used to determine the severity of the stroke [30]. The lower the score, the milder the stroke [30]. As for severity at discharge, the modified Rankin scale (mRS) score was assessed [31,32]. The mRS score is widely used to assess the outcome of AIS and is based on a scale of 0–6 [31,32]. An mRS score of 0 indicates no neurological symptoms, 5 indicates being bedridden from a severe handicap, and 6 denotes death [31,32]. Patients with an mRS score of 2 can live independently, whereas those with a score of 3 cannot live independently [31,32]. In the present study, an mRS score ≥ 3 at discharge was defined as a poor outcome. Patient height was measured after the patient was able to stand. If this was not feasible, the formula described by Mayer et al. [33] was applied. Weight was assessed with the patients in a standing position. If patients could not maintain this position, weight was measured using a wheelchair scale or a suspended scale. If neither method was feasible, the most reliable value from the most recent medical record was used. All patients were evaluated for complications during hospitalization, including pneumonia, urinary tract infection (UTI), and cardiovascular complications, such as acute heart failure, myocardial infarction, pulmonary embolism, and deep vein thrombosis. The present study was approved by the Institutional Research Review Board of Nihon University (approval no: RK-210713-6).

### 2.2. Definition of Overhydration and Sarcopenia

BIA was determined in all patients in the supine resting position using Inbody S10 (InBody Co., Ltd., Seoul, Republic of Korea). BIA was performed immediately after the measurement of height and weight, and the specific days when BIA was performed were recorded. ECW/TBW and muscle mass were calculated by combining BIA, height, and body weight. The estimated ECW/TBW ratio is 0.356–0.403 in healthy adults [11,12,13]. The cutoff ECW/TBW ratio, which defines the state of overhydration, varies across studies, ranging from 0.390 to 0.400 [9,17,34,35,36]. In the present study, to identify even mildly overhydrated patients, an ECW/TBW ratio > 0.390 was used to define patients with overhydration. To avoid statistical confounding, the ECW/TBW ratio multiplied by 100 (%ECW/TBW > 39.0) was used to define overhydration as previously reported [15].

Sarcopenia was assessed according to the 2019 sarcopenia diagnostic criteria defined by the Asian Working Group for Sarcopenia [3]. To prevent the effect of gait disturbance resulting from AIS, grip strength was used to evaluate muscle strength. Grip strength was determined using the non-paralyzed side, and decreased grip strength was defined as <28 kg in men and <18 kg in women [3]. Low muscle mass was defined as <7.0 kg/m^2^ in men and <5.7 kg/m^2^ in women [3]. Patients with both decreased grip strength and low muscle mass were diagnosed with sarcopenia [3].

### 2.3. Definition of Nutritionally At-Risk

In 2018, the Global Leadership Initiative on Malnutrition criteria were proposed as the standard diagnosis of undernutrition [37]. Accordingly, patients considered undernourished with existing nutrition screening tools are diagnosed and classified for undernutrition severity based on weight loss rate, body mass index (BMI), and muscle mass [37]. Because muscle mass was also used as an indicator for sarcopenia in the present study, the geriatric nutritional risk index (GNRI) score [38] was used as a nutrition-related indicator. Nutritional risk, as assessed by the GNRI score, indicates a high risk of complications and death related to undernutrition [38]. GNRI score is associated with stroke Functional Independence Measure gains [19] and outcomes [20,21] and is calculated using the following formula:

$$\begin{array}{cc} GNRI\;score\;= & 14.89\times serum\;albumin\;(g/dL)\\ & +\;41.7\;\times \;body\;weight\;(kg)/ideal\;body\;weight\;(kg) \end{array}$$

A low GNRI of <98 indicates nutritional risk (i.e., nutritionally at-risk) [38]. The ideal body weight (IBW) was defined as a body weight with a BMI of 22.0 kg/m^2^ using the following formula [21]:

$$IBW\;=\;22.0\;\times \;height\;(m^{2})$$

### 2.4. Statistical Analysis

All statistical analyses were performed using SPSS 28.0 software (IBM Corp., Armonk, NY, USA). Fisher’s exact probability test was used for nominal variables and the Mann–Whitney *U* test was used for ordinal and continuous variables. Univariate analysis was performed to compare the good and poor AIS outcome groups. In addition, univariate analyses were performed to compare clinical characteristics between patients with and without overhydration (%ECW/TBW of >39.0 and ≤39.0, respectively), between those with and without nutritional risk (GNRI of ≥98 and <98, respectively), and between those with and without sarcopenia. To determine whether overhydration, being nutritionally at-risk, and sarcopenia impacted the AIS outcome, logistic regression analysis was performed with the outcome as the independent variable and overhydration, being nutritionally at-risk, sarcopenia, and other variables that might impact the outcome as the dependent variables. Variables with a variance inflation factor >10 were excluded. In addition, to evaluate the presence of monotonically increasing or decreasing trends between the groups, the Jonckheere–Terpstra test was used with a 2 × 4 crosstabulation table, including good and poor outcomes as row variables and the number of abnormalities among the overhydration, nutritionally at-risk, and sarcopenia groups as column variables (0–3). The impact on AIS outcomes of both overhydration and low muscle mass was evaluated using Fisher’s exact probability test. Statistical significance was defined as a *p*-value < 0.05.

## 3. Results

### 3.1. Study Cohort

**Figure 1:**
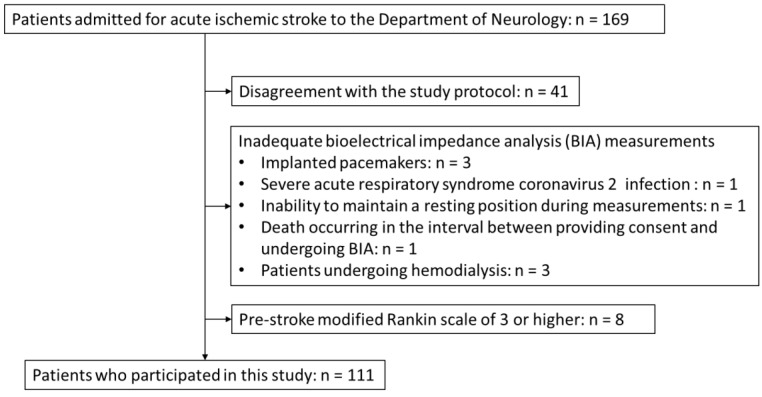
Flowchart depicting patient selection criteria.

Over the study period, 169 patients with AIS were admitted to the Department of Neurology and 58 patients were excluded. The exclusion criteria included disagreement with the study protocol, inadequate BIA, and a pre-AIS mRS score ≥ 3 in 41, 9, and 8 patients, respectively (Figure 1). The final study cohort consisted of 111 patients (109 Japanese and 2 Chinese), including 40 female and 71 male patients, with a median age of 77 (19–99) years. The study dataset is available in Supplemental Material (Table S1).

### 3.2. Univariate Comparison between Two Group

#### 3.2.1. Comparison of the Patients with Good and Poor AIS Outcomes

The characteristics of the patients categorized based on the mRS score are listed in Table 1. Briefly, 43 patients (38.7%), who experienced a poor outcome based on the study definition, were significantly older (median age: 81 vs. 73 years; *p* < 0.001) and had a higher NIHSS score (median score: 3 vs. 1; *p* < 0.001). The number of patients with overhydration and a high %ECW/TBW ratio was significantly higher in the poor outcome group compared to the good outcome group (53.5% vs. 11.8%, *p* < 0.001 and 39.2 vs. 38.2, *p* < 0.001, respectively). The percentage of sarcopenia was 65.1% in the poor outcome group, which was significantly higher compared to that in the good outcome group (23.5%, *p* < 0.001). Moreover, patients in the poor outcome group exhibited lower serum albumin (3.9 vs. 4.2 g/dL, *p* < 0.001) and higher NT-pro-BNP (345.5 vs. 159 pg/mL, *p* < 0.001) compared to those in the good outcome group. However, the percentage of patients with high NT-pro-BNP (40.0% vs. 31.8%, *p* = 0.183) was not significantly different. Being nutritionally at-risk was more common in the poor outcome group compared to the good outcome group (40.0% vs. 11.8%, *p* < 0.001). The complication rates of pneumonia (18.6% vs. 1.8%, *p* = 0.002) and UTI (23.3% vs. 4.4%, *p* = 0.004) during hospitalization and the length of hospital stay were higher in the poor outcome group compared to the good outcome group (21 vs. 12 days, *p* < 0.001).

#### 3.2.2. Comparison of AIS Patients with and without Overhydration

Compared to the patients without overhydration, those with overhydration (*n* = 31, 27.9%) included significantly more female patients (58.1% vs. 27.5%, *p* = 0.003) who were significantly older (83 vs. 74.5 years, *p* < 0.001) and had significantly higher NIHSS scores (3 vs. 1, *p* = 0.005) (Table 2). The percentage of sarcopenia in patients with overhydration was 74.2%, which was significantly higher than in patients without overhydration (26.3%, *p* < 0.001). Blood tests revealed lower serum albumin (3.7 vs. 4.2 g/dL, *p* < 0.001), higher NT-pro-BNP levels (1054 vs. 159 pg/mL, *p* < 0.001), and a higher percentage of high NT-pro-BNP (69.0% vs. 23.3%, *p* < 0.001) in patients with overhydration compared to those without overhydration. Being nutritionally at-risk was significantly more common in patients with overhydration compared to those without overhydration (45.2% vs. 13.8%, *p* < 0.001). For complications during hospitalization, the percentage of UTIs was significantly higher in patients with overhydration compared to those without overhydration (22.6% vs. 7.5%, *p* = 0.034), whereas the rate of pneumonia and cardiovascular complications was not significantly different between the two groups.

#### 3.2.3. Comparison of AIS Patients with and without Nutritional Risk

A comparison of patients with and without nutritional risk is summarized in Table 3. Patients who were nutritionally at-risk (*n* = 25) were older (81 vs. 74 years, *p* = 0.008) and had higher NIHSS (3 vs. 1, *p* = 0.002) scores compared to those who were not nutritionally at-risk (*n* = 86). Physical measurements revealed a lower BMI (20.6 vs. 24.2 kg/m^2^, *p* < 0.001) and higher levels of overhydration (56.0% vs. 19.8%, *p* < 0.001) and sarcopenia (84.0% vs. 26.7%, *p* < 0.001). Blood data revealed higher CRP (0.3 vs. 0.1 md/dL, *p* = 0.002) and NT-pro-BNP (551 vs. 162 pg/mL, *p* < 0.001). The percentage of patients with high NT-pro-BNP was higher in patients who were nutritionally at-risk (56.5% vs. 30.1%, *p* = 0.019). Although there were no significant differences in the rate of complications, such as pneumonia, UTI, and cardiovascular complications, there was a higher rate of poor outcomes (68.0% vs. 30.2%, *p* < 0.001) and longer hospital stays (18 vs. 14 days, *p* = 0.009).

#### 3.2.4. Comparison of AIS Patients with and without Sarcopenia

A comparison of patients with and without sarcopenia is listed in Table 4. Patients with sarcopenia (*n* = 44, 39.6%) were significantly older (81 vs. 72 years, *p* < 0.001) and had higher NIHSS scores (3 vs. 1, *p* = 0.001), lower BMIs (21.7 vs. 24.2 kg/m^2^, *p* < 0.001), and higher %ECW/TBW ratios (39.2 vs. 38.1, *p* < 0.001). NT-pro-BNP levels were significantly higher and the GNRI scores were significantly lower in the sarcopenia group than in the non-sarcopenia group (406 vs. 159 pg/mL, *p* = 0.001 and 98.5 vs. 107.3, *p* < 0.001, respectively). However, the percentage of patients with high NT-pro-BNP was not significant (46.3% vs. 29.2%, *p* = 0.057). In addition, the percentage of nutritionally at-risk patients and pneumonia occurring during hospitalization was significantly higher in the sarcopenia group than in the non-sarcopenia group (47.7% vs. 6.0%, *p* < 0.001, 18.4% vs. 2.7%, *p* = 0.019, respectively).

### 3.3. Relationship between the Number of Comorbidities and AIS Outcome

Table 5 shows the number of comorbidities (i.e., overhydration, being nutritionally at-risk, and sarcopenia) in the good and poor outcome groups. Briefly, 67.6%, 22.1%, 5.9%, and 4.4% of the patients in the good outcome group and 23.3%, 18.6%, 34.9%, and 23.3% of the patients in the poor outcome group had 0, 1, 2, and 3 comorbidities, respectively. The Jonckheere–Terpstra test indicated a significant monotonic trend in the impact of the number of comorbidities across the groups, based on a J-value of 2268.000, with a standardized z-score of 5.273 and a two-tailed *p*-value < 0.001. These results indicate that the number of comorbidities increased monotonically with the poor outcome.

### 3.4. Multivariate Analysis with Outcome as the Dependent Variable

Logistic regression analysis was performed with the good and poor outcome groups as dependent variables and age, sex, NIHSS score, NT-pro-BNP, overhydration, nutritionally at-risk, and sarcopenia as independent variables (Table 6). The analysis revealed age [odds ratio (OR) 1.062, 95% confidence interval (CI) 1.010–1.117; *p* = 0.020], NIHSS score (OR 1.790, 95%CI 1.307–2.451; *p* < 0.001), and overhydration (OR 5.504, 95%CI 1.717–17.648; *p* = 0.001) as independent predictors of poor outcome. The *p*-value for this model was 0.219 using the Hosmer–Lemeshow test. In a sensitivity analysis, the model was analyzed with BMI added as an independent variable, albumin instead of nutritionally at-risk, and decreased grip strength and low muscle mass (present vs. absent) instead of sarcopenia, which yielded similar results.

### 3.5. Relationship between Body Water Balance and Muscle Mass

**Figure 2:**
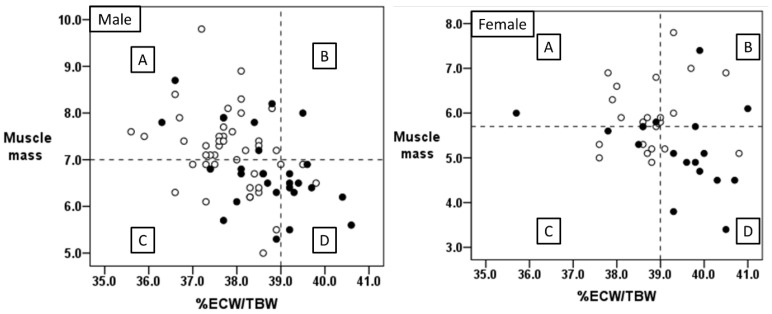
Scatter plots showing the distribution of groups categorized according to %ECW/TBW and muscle mass in patients with AIS. Group A—low %ECW/TBW and high muscle mass; Group B—high %ECW/TBW and high muscle mass; Group C—low %ECW/TBW and low muscle mass; and Group D—high %ECW/TBW and low muscle mass. %ECW/TBW represents the ratio of extracellular fluid to total body water multiplied by 100. White and black circles indicate patients with good and poor outcomes, respectively.

Figure 2 shows the scatter plots of the relationship between %ECW/TBW and muscle mass. The patients were separated into the following four groups based on the ECW/TBW ratio and muscle mass using a %ECW/TBW cutoff value of 39.0 and muscle mass cutoff values of 7.0 kg/m^2^ in men and 5.7 kg/m^2^ in women [3]: Group A—low %ECW/TBW and high muscle mass; Group B—high %ECW/TBW and high muscle mass; Group C—low %ECW/TBW and low muscle mass; and Group D—high %ECW/TBW and low muscle mass. The overall cohort included 71 male and 40 female patients. Groups A, B, C, and D included 34 (30.6%), 1 (0.9%), 24 (21.6%), and 12 (10.8%) male patients, respectively, and 14 (35%), 7 (17.5%), 8 (20%), and 11 (27.5%) female patients, respectively.

The relationship between outcomes and the four groups was analyzed. In Group A, 39 patients showed good outcomes and nine had poor outcomes. There were four of each in Group B, 21 with good and 11 with poor outcomes in Group C, and 4 and 19 in Group D, respectively. As shown in Table 7, the ratio of patients with poor outcomes was higher in Group D (patients with low muscle mass and high %ECW/TBW) than in the other groups (*p* < 0.001) based on a sensitivity of 0.442, specificity of 0.941, positive predictive value of 0.826, negative predictive value of 0.727, positive likelihood ratio of 7.512, and positive likelihood ratio of 0.593, respectively, predicting poor AIS outcome.

## 4. Discussion

In the present study, we examined patients with AIS living independently before AIS onset. We evaluated the contribution of abnormal body water balance, particularly overhydration, nutritional risk, and sarcopenia to poor outcomes upon discharge. Although these three factors are interrelated [16,17,18], the results suggest that the higher the overlap among these factors, the higher the rate of poor outcomes. In addition, the concomitant presence of overhydration and low muscle mass was highly specific for poor outcomes following AIS. Our multivariate analysis revealed that overhydration, but not nutritional risk or sarcopenia, was a predictor of poor outcomes, independent of age and stroke severity. This study showed that BIA can be measured early after admission and is a potentially useful test for predicting the outcome of AIS.

**Figure 3:**
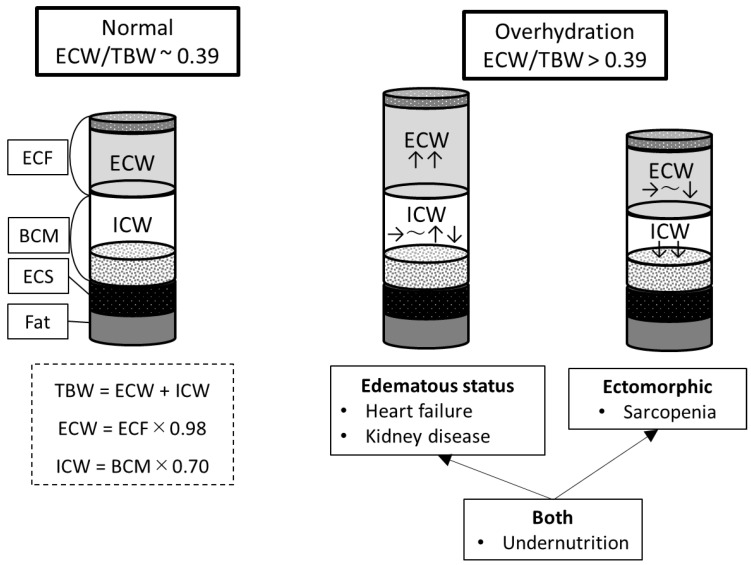
A model describing body composition and overhydration. BCM, body cell mass; ECF, extracellular fluid; ECSs, extracellular solids; ECW, extracellular water; ICW, intracellular water; and TBW, total body water.

First, with respect to body composition and overhydration, the human body consists of four major components: fat, extracellular solids (ECSs), extracellular fluid (ECF), and body cell mass (BCM), as illustrated in Figure 3 [8,39].

Among the four types of body components, fat consists of approximately 75% lipids, with only a small amount of water [40]. Unless the patient is morbidly obese or has undergone rapid weight change [28,41], the effect of fat water content on body water balance is minimal. ECSs form a non-metabolizable component consisting of organic and inorganic compounds, such as bone matrix, collagen, and reticular and elastic fibers [8,42]. ECSs do not contribute to alterations in water balance as the ECS/TBW ratio is constant [8,42]. Conversely, there are two important factors associated with body water balance: ECW and ICW. ECF represents the fluid in the circulating plasma and the interstitium [43]. Approximately 98% of the ECF is water mass, which is represented by ECW [8]. On the other hand, BCM is primarily composed of water and protein. Water mass accounts for approximately 70% of the BCM, which is represented by ICW [8]. Cells are sensitive to changes in water content and cell swelling and shrinkage because osmotic changes are associated with water movement to and from the ECW [8,39,44]. This mechanism maintains the ICW/ECW ratio within a certain range in the event of water imbalance, whereas its failure leads to abnormalities in water balance [8,39,44]. Thus, the ECW and ICW represent body compartments that allow dynamic changes in water balance. Two pathogenic mechanisms should be considered in the interpretation of an elevated ECW/TBW ratio. TBW is calculated as the sum of ECW and ICW; therefore, an increase in the ECW/TBW ratio can be ectomorphic, whereas a decrease in ICW is greater than a decrease in ECW. In edematous states, ECW increases, while ICW remains unchanged or changes slightly (Figure 2) [39]. The extent to which ectomorphic and edematous states are involved varies depending on the disorder. For example, the edematous state (i.e., increased ECW) plays a more significant role in the increased ECW/TBW ratio in patients with acute heart failure [36] and kidney disease [35]; however, the involvement of the two pathogenic mechanisms in AIS patients has not been determined.

Second, the causes of overhydration observed in AIS patients with poor outcomes in this study must be considered. Previous studies using BIA in ischemic and/or hemorrhagic stroke patients have speculated that abnormalities in body water balance in these patients include muscle damage due to stroke-induced catabolism [23], cerebral edema [24,25], and inflammatory processes as a systemic response to stroke [25]. In this study, because the BIA device placed electrodes at the extremities and measured limb and trunk impedance, it is unlikely that the values obtained were affected by cerebral edema. In addition, there was no significant difference in CRP levels between the presence and absence of overhydration, which suggests that overhydration was not caused by inflammation. As discussed above, one cause of overhydration is ectomorphic pathology (Figure 3). Skeletal muscle mass accounts for nearly half the mass of fat-free mass (ECF + BCM + ECS) in the human body [8]. Approximately 79% of the weight of skeletal muscle is accounted for by water mass, which belongs to the ICW [8]. Therefore, loss of muscle mass results in a decrease in ICW and is related to sarcopenia [3,4]. The prevalence of sarcopenia was higher in patients with a higher ECW/TBW ratio in the present study, which indicates that it accompanies AIS and may contribute to overhydration. Clinical conditions with edema, such as heart failure [36] and kidney disease causing volume overload [35], are other causes of overhydration. In the present study, because creatinine was not significantly different between patients with and without overhydration, kidney disease involvement was negative. On the other hand, NT-pro-BNP levels were higher in patients with overhydration, which indicated that mild heart failure may have partially affected overhydration. Patients with poor outcomes, overhydration, and sarcopenia exhibited lower GNRI scores and a higher probability of being nutritionally at-risk. Low albumin and low BMI are associated with protein-energy undernutrition (PEU) [45]. PEU results in bradycardia, low cardiac output, impaired tissue perfusion, capillary leakage, and, ultimately, edema [45]. The results of this study indicate that poor outcomes and overhydration may be related to an edematous status because of PEU. In summary, the pathogenesis of overhydration observed in poor outcome patients with AIS is influenced by edema to some extent because of low protein and heart failure, in addition to decreased ICW resulting from sarcopenia. In other words, the increase in the ECW/TBW ratio may be related to both ectomorphism and an edematous state. Indeed, multivariate analysis showed a stronger association with outcome in the overhydration group, which included an assessment of both ectomorphism and an edematous state, compared to sarcopenia or being nutritionally at-risk. In addition, the high positive predictive value of a high ECW/TBW ratio and a low muscle mass (Group D, Figure 3) for poor outcome suggests that the measurement of muscle mass and the ECW/TBW ratio using BIA may be useful in predicting patient outcomes after AIS in an acute care hospital.

Finally, the present study findings have several implications for patient care. A study conducted in rehabilitation units revealed that most patients with stroke have poor nutritional status and experience muscle mass loss during the acute phase of stroke [19]. Therefore, high energy and protein intake, in addition to sufficient rehabilitation duration, are recommended [19,22]. Nutritional management and rehabilitation are also critical to prevent sarcopenia [3]. In the present study, the rate of patients with poor outcomes was higher among patients with overhydration, nutritional risk, and sarcopenia at the time of hospitalization. Therefore, future studies should evaluate the utility of nutritional management and rehabilitation for comorbid overhydration, nutritional risk, and sarcopenia in patients with AIS.

This study has several limitations. First, this was a single-center study with a limited number of patients. Because this study prospectively enrolled eligible patients who were consecutively hospitalized during the study period, power analysis was not performed. Second, ECW and TBW, measured by BIA, differ slightly by race and ethnicity [28,46]. In the present study, most of the patients were Japanese, with a few Chinese patients. Thus, the diagnostic criteria for sarcopenia used in Asia were considered in the analyses. Future studies should determine whether our findings are comparable to those for other ethnic groups or those based on different sarcopenia diagnostic criteria. Third, the diets and the intravenous fluids administered to patients with AIS after admission were not evaluated. Assessing patients on specific days or following hydration after hospitalization may provide further information in this regard.

## 5. Conclusions

AIS patients with overhydration, being nutritionally at-risk and sarcopenia had poor outcomes. The higher the overlap of these three factors, the higher the rate of poor outcomes. Patients showing both overhydration and low muscle mass had a particularly high rate of poor outcomes. Overhydration, rather than sarcopenia or nutritional risk, was associated with poor outcomes according to multivariable analysis. Assessing body composition with BIA was useful in predicting outcomes after AIS in an acute care hospital.

## Figures and Tables

**Table 1:** Comparison of the patients with good and poor AIS outcomes.

	Total (*n* = 111)	Good Outcome (mRS Score < 3) (*n* = 68)	Poor Outcome (mRS Score ≥ 3) (*n* = 43)	*p*-Value
Sex (female, %)	40 (36.0%)	23 (33.8%)	17 (39.5%)	0.341
Age (years)	77 (19–99)	73 (19–90)	81 (51–99)	<0.001 *
NIHSS score	2 (0–30)	1 (0–7)	3 (0–30)	<0.001 *
Body measurements				
Height (cm)	161 (135–180)	163 (135–177)	161 (135–180)	0.277
Body weight (kg)	60 (34–109)	61 (34–109)	58 (35–80)	0.092
BMI (kg/m^2^)	23.5 (14.3–37.7)	23.9 (18.2–37.7)	23.1 (14.3–29.8)	0.263
%ECW/TBW	38.5 (35.6–41.0)	38.2 (35.6–40.8)	39.2 (35.7–41.0)	<0.001 *
%ECW/TBW > 0.390	31 (27.9%)	8 (11.8%)	23 (53.5%)	<0.001 *
Muscle mass (kg/m^2^)	6.5 (3.4–9.8)	6.9 (4.9–9.8)	6.2 (3.4–8.7)	0.005 *
Grip strength (kg)	22 (0–49)	25.5 (0–49)	15 (0–42)	<0.001 *
Sarcopenia	38 (34.2%)	16 (23.5%)	28 (65.1%)	<0.001 *
Days from admission to evaluation ^1^	8 (1–24)	7 (1–15)	8 (0–24)	0.034 *
Laboratory data				
Albumin (g/dL)	4.1 (2.7–5.0)	4.2 (3.3–5)	3.9 (2.7–5)	<0.001 *
Creatinine (mg/dL)	0.9 (0.5–10.1)	0.9 (0.5–10.1)	0.8 (0.5–4.6)	0.910
CRP (mg/dL)	0.1 (0.1–10.0)	0.1 (0.1–3.9)	0.2 (0.1–10)	0.202
NT-pro-BNP (pg/mL)	221.5 (10–9463)	159 (10–7099)	345.5 (34–9463)	0.006 *
NT-pro-BNP > 500 pg/mL	38 (35.8%)	21 (31.8%)	17 (40.0%)	0.183
HbA1c (%)	6.0 (4.8–10.8)	6.0 (4.8–10.3)	6.2 (5.1–10.8)	0.242
GNRI score	104.7 (76.4–135.5)	106.7 (84.5–135.5)	100.9 (76.4–124.8)	<0.001 *
Nutritionally at-risk ^2^	25 (22.5%)	8 (11.8%)	17 (40.0%)	<0.001 *
Complications				
Pneumonia	9 (8.1%)	1 (1.5%)	8 (18.6%)	0.002 *
Urinary tract infection	13 (11.7%)	3 (4.4%)	10 (23.3%)	0.004 *
Cardiovascular	5 (4.5%)	2 (2.9%)	3 (7.0%)	0.292
Clinical course				
Length of hospitalization (days)	15 (4–67)	12 (4–51)	21 (12–67)	<0.001 *

Continuous data are presented as medians (range). * *p* < 0.05, ^1^ day 0 as the date of admission, and ^2^ GNRI < 98. BMI, body mass index; CRP, *C*-reactive protein; ECW, extracellular water; GNRI, geriatric nutritional risk index; HbA1c, glycated hemoglobin; NIHSS, National Institutes of Health stroke scale; NT-pro-BNP, *N*-terminal–pro-brain natriuretic peptide; and TBW, total body water.

**Table 2:** Comparison of AIS patients with and without overhydration.

	Normal (*n* = 80)	Overhydration (*n* = 31)	*p*-Value
Sex (female, %)	22 (27.5%)	18 (58.1%)	0.003 *
Age (years)	74.5 (19–95)	83 (48–99)	<0.001 *
NIHSS score	1 (0–18)	3 (0–30)	0.005 *
Body measurements			
Height (cm)	164 (139–180)	158 (135–175)	0.025 *
Body weight (kg)	61 (39–109)	54 (34–77)	0.011 *
BMI (kg/m^2^)	24.1 (14.3–37.7)	22.5 (16.7–33.8)	0.054
Muscle mass (kg/m^2^)	6.8 (4.9–9.8)	6.1 (3.4–8)	0.001 *
Grip strength (kg)	25.5 (0–49)	11 (0–32)	<0.001 *
Sarcopenia	21 (26.3%)	23 (74.2%)	<0.001 *
Days from admission to evaluation ^1^	7 (1–21)	8 (0–24)	0.108
Laboratory data			
Albumin (g/dL)	4.2 (3.4–5)	3.7 (2.7–4.8)	<0.001 *
Creatinine (mg/dL)	0.9 (0.5–10.1)	0.8 (0.5–4.6)	0.541
CRP (mg/dL)	0.1 (0.1–3.9)	0.1 (0.1–10)	0.253
NT-pro-BNP (pg/mL)	159 (10–9463)	1054 (97–6581)	<0.001 *
NT-pro-BNP > 500 pg/mL	18 (23.3%)	20 (69.0%)	<0.001 *
HbA1c (%)	6.0 (4.8–10.3)	6.2 (5.3–10.8)	0.074
GNRI score	106.0 (88.1–135.5)	98.9 (76.4–129.5)	<0.001 *
Nutritionally at-risk ^2^	11 (13.8%)	14 (45.2%)	<0.001 *
Complications			
Pneumonia	5 (6.3%)	4 (12.9%)	0.217
Urinary tract infection	6 (7.5%)	7 (22.6%)	0.034 *
Cardiovascular	2 (2.5%)	3 (9.7%)	0.132
Clinical course			
Length of hospitalization (days)	14 (4–67)	18 (10–51)	<0.001 *
Poor prognosis	20 (25.0%)	23 (74.2%)	<0.001 *

Continuous data are presented as medians (range). * *p* < 0.05, ^1^ day 0 as the date of admission, and ^2^ GNRI < 98. BMI, body mass index; CRP, *C*-reactive protein; ECW, extracellular water; GNRI, geriatric nutritional risk index; HbA1c, glycated hemoglobin; NIHSS, National Institutes of Health stroke scale; NT-pro-BNP, *N*-terminal–pro-brain natriuretic peptide; and TBW, total body water.

**Table 3:** Comparison of AIS patients with and without nutritional risk.

	Not Nutritionally At-Risk (*n* = 86)	Nutritionally At-Risk (*n* = 25)	*p*-Value
Sex (female, %)	29 (33.7%)	11 (44.0%)	0.355
Age (years)	74 (19–96)	81 (48–99)	0.008 *
NIHSS score	1 (0–30)	3 (1–19)	0.002 *
Body measurements			
Height (cm)	163 (138.3–180)	159.3 (135–175)	0.037 *
Body weight (kg)	63 (42.5–109)	50 (34–63.9)	<0.001 *
BMI (kg/m^2^)	24.2 (19.2–37.7)	20.6 (14.3–24.3)	<0.001 *
%ECW/TBW	38.3 (35.7–40.5)	39.4 (37.6–41.0)	<0.001 *
%ECW/TBW > 0.390	17 (19.8%)	14 (56.0%)	<0.001 *
Muscle mass (kg/m^2^)	6.9 (3.8–9.8)	5.6 (3.4–6.9)	<0.001 *
Grip strength (kg)	25 (0–49)	12 (0–31)	<0.001 *
Sarcopenia	23 (26.7%)	21 (84.0%)	<0.001 *
Days from admission to evaluation ^1^	8 (0–21)	7 (2–24)	0.406
Laboratory data			
Albumin (g/dL)	4.2 (3.4–5.0)	3.6 (2.7–4.1)	<0.001 *
Creatinine (mg/dL)	0.9 (0.49–2.12)	0.9 (0.49–10.1)	0.382
CRP (mg/dL)	0.1 (0.1–8.2)	0.3 (0.1–10)	0.002 *
NT-pro-BNP (pg/mL)	162 (10–3916)	551 (133–9463)	<0.001 *
NT-pro-BNP > 500 pg/mL	25 (30.1%)	13 (56.5%)	0.019 *
HbA1c (%)	6 (4.8–10.3)	5.8 (5.2–8.6)	0.935
GNRI score	106.7 (98.3–136.5)	93.6 (76.4–97.8)	<0.001 *
Complications			
Pneumonia	6 (7.0%)	3 (12%)	0.419
Urinary tract infection	7 (8.1%)	6 (24%)	0.070
Cardiovascular	3 (3.5%)	2 (8.0%)	0.314
Clinical course			
Length of hospitalization (days)	14 (4–51)	18 (7–67)	0.009 *
Poor prognosis	26 (30.2%)	17 (68.0%)	<0.001 *

Continuous data are presented as medians (range). * *p* < 0.05 and ^1^ day 0 as the date of admission. BMI, body mass index; CRP, *C*-reactive protein; ECW, extracellular water; GNRI, geriatric nutritional risk index; HbA1c, glycated hemoglobin; NIHSS, National Institutes of Health stroke scale; NT-pro-BNP, *N*-terminal–pro-brain natriuretic peptide; and TBW, total body water.

**Table 4:** Comparison of AIS patients with and without sarcopenia.

	Non-Sarcopenia (*n* = 67)	Sarcopenia (*n* = 44)	*p*-Value
Sex (female, %)	24 (35.8%)	16 (36.4%)	0.556
Age (years)	72 (19–95)	81 (31–99)	<0.001 *
NIHSS score	1 (0–18)	3 (0–30)	0.001 *
Body measurements			
Height (cm)	164 (139–180)	159 (135–175)	0.028 *
Body weight (kg)	64 (43–109)	53 (34–72)	<0.001 *
BMI (kg/m^2^)	24.2 (19.0–37.7)	21.7 (14.3–26.6)	<0.001 *
%ECW/TBW	38.1 (35.6–41.0)	39.2 (36.6–40.8)	<0.001 *
%ECW/TBW > 0.390	8 (11.9%)	23 (52.3%)	<0.001 *
Muscle mass (kg/m^2^)	7.1 (5.1–9.8)	5.9 (3.4–6.9)	<0.001 *
Grip strength (kg)	29 (0–49)	16 (0–27)	<0.001 *
Days from admission to evaluation ^1^	7 (1–21)	8 (0–24)	0.058
Laboratory data			
Albumin (g/dL)	4.2 (3.4–5)	3.9 (2.7–5)	<0.001 *
Creatinine (mg/dL)	0.9 (0.5–10.1)	0.9 (0.5–4.6)	0.787
CRP (mg/dL)	0.1 (0.1–3)	0.2 (0.1–10)	0.080
NT-pro-BNP (pg/mL)	159 (10–7099)	406 (34–9463)	0.001 *
NT-pro-BNP > 500 pg/mL	19 (29.2%)	19 (46.3%)	0.057
HbA1c (%)	5.9 (4.8–10.3)	6.2 (5.3–10.8)	0.323
GNRI score	107.3 (93.3–135.5)	98.5 (76.4–124.8)	<0.001 *
Nutritionally at-risk ^2^	4 (6.0%)	21 (47.7%)	<0.001 *
Complications			
Pneumonia	2 (3%)	7 (15.9%)	0.019 *
Urinary tract infection	5 (7.5%)	8 (18.2%)	0.080
Cardiovascular	4 (6%)	1 (2.3%)	0.338
Clinical course			
Length of hospitalization (days)	13 (6–51)	18 (4–67)	0.001 *
Poor prognosis	15 (22.4%)	28 (63.6%)	<0.001 *

Continuous data are presented as medians (range); * *p* < 0.05, ^1^ day 0 as the date of admission, and ^2^ GNRI < 98. BMI, body mass index; CRP, *C*-reactive protein; ECW, extracellular water; GNRI, geriatric nutritional risk index; HbA1c, glycated hemoglobin; NIHSS, National Institutes of Health stroke scale; NT-pro-BNP, *N*-terminal–pro-brain natriuretic peptide; and TBW, total body water.

**Table 5:** Inter-relationships between overhydration, being nutritionally at-risk, and sarcopenia in patients with AIS.

Number of Comorbidities	Comorbidities	Good Outcome (*n* = 68)	Poor Outcome (*n* = 43)	*p*-Value
0	None	46 (67.6%)	10 (23.3%)	<0.001 *
1	Overhydration	4	3	
	Being nutritionally at-risk	2	1	
	Sarcopenia	9	4	
	Total	15 (22.1%)	8 (18.6%)	
2	Overhydration + being nutritionally at-risk	0	1	
	Overhydration + sarcopenia	1	9	
	Being nutritionally at-risk + sarcopenia	3	5	
	Total	4 (5.9%)	15 (34.9%)	
3	All	3 (4.4%)	10 (23.3%)	

* *p* < 0.05.

**Table 6:** Multivariate analysis with good and poor AIS outcome groups as objective variables.

	B	Standard Error	*p*-Value	Exp(B)	95% Confidence Interval
Age	0.060	0.026	0.020 *	1.062	1.010–1.117
NIHSS on admission	0.582	0.160	<0.001 *	1.790	1.307–2.451
Overhydration	1.705	0.594	0.004 *	5.504	1.717–17.648

Other variables, such as sex, NT-pro-BNP levels, being nutritionally at-risk, and sarcopenia, which did not exhibit significant effects in analyses, were excluded from the model. * *p* < 0.05.

**Table 7:** Relationship between overhydration and low muscle mass as comorbidities with AIS outcome.

	Good Outcome (*n* = 68)	Poor Outcome (*n* = 43)	*p*-Value
Group A (normal %ECW/TBW and muscle mass)	39	9	
Group B (high %ECW/TBW and normal muscle mass)	4	4	
Group C (normal %ECW/TBW and low muscle mass)	21	11	
Non-Group D	64	24	*p* < 0.001 *
Group D (high %ECW/TBW and low muscle mass)	4	19	

%ECW/TBW: percentage of extracellular water to total body water. * *p* < 0.05.

## Data Availability

The datasets used in this study are available in the Supplemental Material. The other data used during the current study are available from the corresponding author upon reasonable request.

## Supplementary Materials

The following supporting information can be downloaded at: https://www.mdpi.com/article/10.3390/nu16132165/s1, Table S1: Dataset.
